# VATS right apical segmentectomy for lung cancer in a patient with tracheal bronchus

**DOI:** 10.1016/j.ijscr.2021.106007

**Published:** 2021-05-20

**Authors:** Takaki Akamine, Takuro Kometani, Naoko Miura, Hayashi Yoshimura, Yasunori Shikada

**Affiliations:** a Department of Surgery, Saiseikai Fukuoka General Hospital, 1-3-46 Tenjin, Chuo-ku, Fukuoka 810-0001, Japan; b Department of Anesthesia, Saiseikai Fukuoka General Hospital, 1-3-46 Tenjin, Chuo-ku, Fukuoka 810-0001, Japan

**Keywords:** Tracheal bronchus, Lung cancer, Segmentectomy, Case report

## Abstract

**Introduction:**

A tracheal bronchus is rarely observed, occurring in only 1% of all patients who undergo thoracic surgeries. We rarely encounter lung cancer in a patient with a tracheal bronchus; however, it is essential to know the distinctive perioperative management strategy for patients with a tracheal bronchus.

**Case presentation:**

We report a 72-year-old man with lung cancer located in the right apical segment supplied by a tracheal bronchus. Annual chest computed tomography performed as follow-up after colon cancer resection showed an enlarging pulmonary nodule with pure ground-glass opacity, which was suspected to be lung adenocarcinoma. The nodule was located in the right apical segment. The apical segment was independently supplied by a single pulmonary artery superior trunk and a tracheal bronchus that branched directly from the trachea at 1.2 cm above the carina. The pulmonary vein branching pattern was uncommon in that the central vein that usually runs through B2 (posterior bronchus) and B3 (anterior bronchus) was missing. The patient underwent video-assisted thoracoscopic apical segmentectomy under one-lung ventilation using a left-sided double-lumen tube.

**Discussion:**

Anomalous venous return accompanied with tracheal bronchus has been described in some reports. Since pulmonary vein is important during segmentectomy, the surgeon should pay particular attention to the venous return.

**Conclusion:**

Preoperative three-dimensional graphic imagery helped us accurately identify the anatomical anomaly to enable the successful segmentectomy in a patient with a tracheal bronchus. We review the relevant literature regarding the perioperative management of patients with a tracheal bronchus.

## Highlights

• A tracheal bronchus is rarely observed in patients who undergo thoracic surgeries.
• Be aware of anomalous venous return accompanied with tracheal bronchus.
• Tracheal cuff of a double lumen tube may obstruct the tracheal bronchus.
• Distance from carina to tracheal bronchus is important for successful anesthesia.
• We review the perioperative management of patients with a tracheal bronchus.

## 1 Introduction

A tracheal bronchus is a rare congenital anomaly in which a bronchus originates directly from the lateral wall of the trachea and connects to the upper lobe [1]. The prevalence of a tracheal bronchus is reported to be 0.1% to 2% [1]. Generally, a tracheal bronchus is detected incidentally in asymptomatic patients by bronchoscopic or radiologic examinations. A recent cohort study reported that the incidence of a tracheal bronchus in all patients undergoing thoracic surgery was 1.08% (77 of 7102 patients) [2]. Therefore, surgeons seldom resect lung cancer from a segment supplied by a tracheal bronchus [3]. Nevertheless, it is essential to preoperatively identify such bronchial anomalies to enable the selection of the optimal perioperative management strategy. We report a case in which video-assisted thoracoscopic surgery (VATS) segmentectomy was performed to resect lung cancer located in the right apical segment supplied by a tracheal bronchus, and review the relevant literature regarding the perioperative management of patients with a tracheal bronchus. This paper has been reported in line with the SCARE 2020 criteria [4].

## 2 Case report

**Fig. 1:**
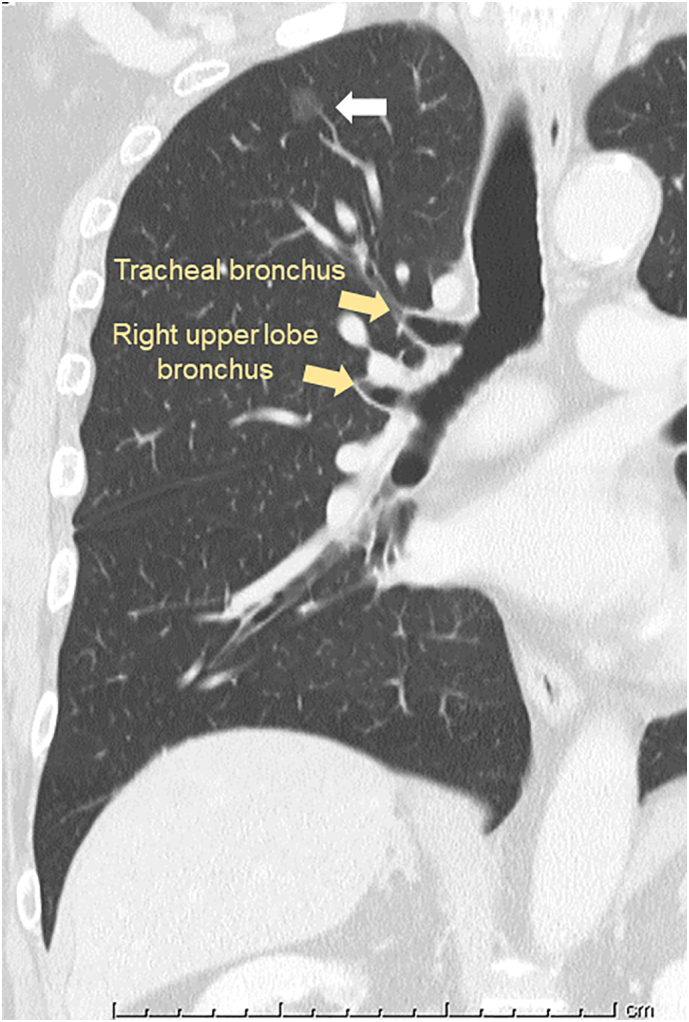
Preoperative coronal computed tomography image. A pure ground-glass opacity nodule (white arrow) is located in the right apical segment that is supplied by a tracheal bronchus.

**Fig. 2:**
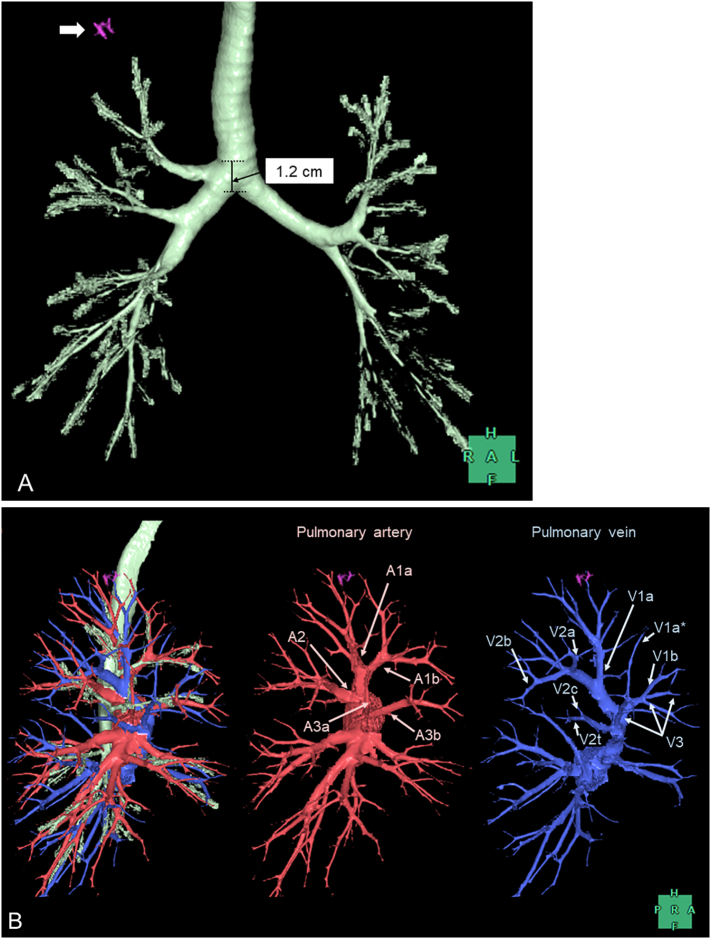
Three-dimensional computed tomography image. (A) The tracheal bronchus of the right apical segment branches from the trachea within 1.2 cm above the carina. The pink nodule (white arrow) is the tumor. (B) The pulmonary vein runs ventral to the pulmonary artery. The superior trunk (A1) independently branches from the main pulmonary artery. V1a and V2a + b share a common trunk that drains to the anterior vein, while the small branch of V1a (V1a*) drains to V1b.

**Supplementary Fig. 1:**
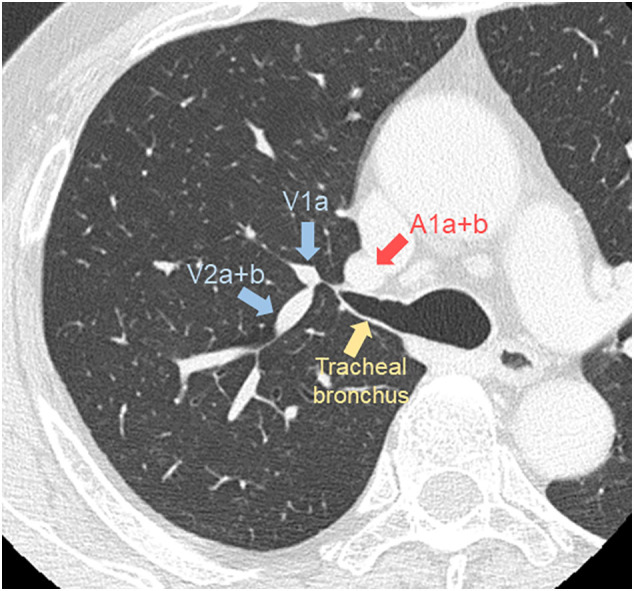
Axial CT slice at the level of the tracheal bronchus demonstrating the locations of A1a + b, V1a, and V2a + b.

The patient was a 72-year-old man who had never smoked and was receiving annual follow-up after curative resection of colon cancer (pT3N0M0, Stage II) with no recurrence. Chest computed tomography (CT) performed 4 years previously had revealed a 4-mm-diameter nodule with pure ground-glass opacity. Recent CT showed that the nodule had increased to 8 mm in diameter. Therefore, the lesion was suspected to be a primary lung cancer, and he was referred to our hospital for surgery. CT showed that the nodule was located in the right apical segment, which had a bronchus that directly originated from the trachea 1.2 cm above the carina (Fig. 1, Fig. 2A). The right upper lobe bronchus bifurcated to the anterior (S3) and posterior (S2) segments. Reconstructed three-dimensional (3D)-CT and an axial CT image taken at the level of the tracheal bronchus revealed that the apical segmental artery (A1) independently branched from the main pulmonary artery (PA), and V1a and V2a + b shared a common trunk that drained to the anterior vein (Fig. 2B, Supplementary Fig. 1).

**Fig. 3:**
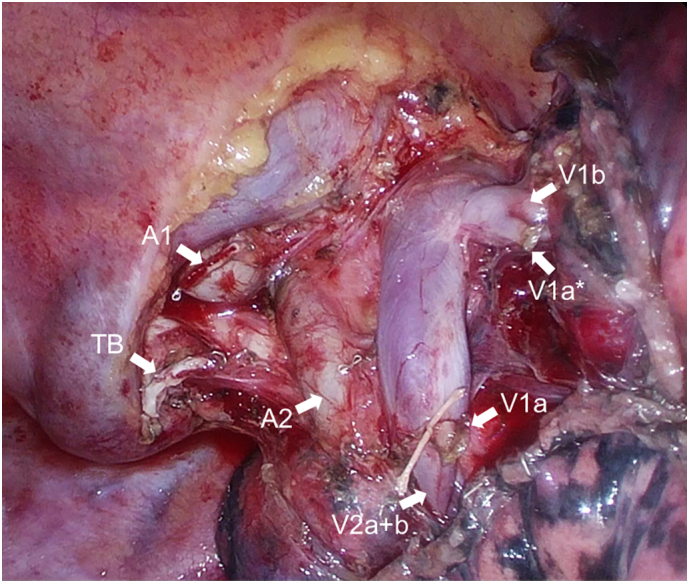
Intraoperative view after video-assisted thoracic segmentectomy. The photograph shows the stump of A1, V1a, small branch of V1a (V1a*), and the tracheal bronchus (TB).

The patient was intubated with a left-sided double-lumen tube (DLT; 37Fr, Mallinckrodt™, Covidien, Ireland) under fiberoptic bronchoscopy. The tracheal bronchus was observed directly under the tracheal cuff. Video-assisted thoracic apical segmentectomy under one-lung ventilation was performed via four incisions: a 3.0-cm incision at the middle axillary line at the 5th intercostal space (ICS), a 15-mm incision at the anterior axillary line at the 4th ICS for the second assistant, a 12-mm incision at the anterior axillary line at the 3rd ICS for the camera, and a 7-mm incision at the posterior axillary line at the 4th ICS. The apical segmental artery (A1) was cut, and the bifurcation of the tracheal bronchus (B1) and the remaining bronchi (B2 + B3) was identified. The tracheal bronchus (B1) was isolated and cut using an endostapler. V1a was ligated and cut while preserving V2a + b. A small branch of V1a (V1a*) draining to V1b was cut using an ultrasonic energy device (Fig. 3). The indocyanine green fluorescence method was used to visualize the intersegmental border [5]. Right apical segmentectomy was completed using the endostapler (Fig. 3). Histopathological examination revealed adenocarcinoma in situ. The postoperative course was uneventful, and the patient was discharged 5 days postoperatively. At one-year follow-up, the patient had no evidence of radiographic recurrence.

## 3 Discussion

The three main types of tracheal bronchus are displaced, supernumerary, and true [3]. The displaced type coexists with a bifurcated upper lobe bronchus, with an anomalous tracheal bronchus taking the place of the missing bronchus. A supernumerary tracheal bronchus coexists with a normal trifurcated upper lobe bronchus. A true tracheal bronchus is when the entire trifurcated upper lobe bronchus originates from the supracarinal trachea. The most common type is the displaced tracheal bronchus, comprising approximately 80% of all tracheal bronchi and typically involving displacement of the apical segment bronchus [6], as in the present case. Fortunately, the lung cancer was a pure ground-glass opacity nodule that was suspected to be adenocarcinoma in situ, suggesting that sublobar resection such as wedge resection or segmentectomy was an acceptable procedure [7]. Moreover, the nodule was located in the apical segment with a displaced tracheal bronchus. To our knowledge, this is the first report of VATS segmentectomy of an apical segment with a displaced tracheal bronchus.

Successful surgery in a patient with a tracheal bronchus requires the preoperative identification of any anatomical anomalies accompanying the tracheal bronchus. A tracheal bronchus may be associated with anomalous venous return in which branches of the upper pulmonary vein travel behind the main PA [[8], [9], [10]]. Nakamura et al. reported a case in which the upper lobe branch of the pulmonary vein (V1–3) ran dorsal to the PA, while V2t and V2c ran ventral to the PA [8]. Qi et al. reported a case in which the V1 ran dorsal to the PA, while the other veins were ventral as usual [9]. The pulmonary vein anomaly can be identified effectively by using 3D-CT [11,12]. In addition, preoperative 3D-CT image assessment of the pulmonary vessels aids in the performance of safe VATS anatomical resection [13]. Therefore, careful preoperative examination of the anatomy, especially regarding the pulmonary venous return, is essential for anatomical resection in a patient with a tracheal bronchus. Although there was no such anomalous venous return in the present case, the pulmonary vein branching pattern was uncommon in that the central vein that usually runs through B2 (posterior bronchus) and B3 (anterior bronchus) was missing. Moreover, the right upper pulmonary vein pattern in the present case did not belong to any of the four types reported by Shimizu et al. [12]. Thus, recognition of the anatomical anomaly based on 3D-CT imagery help us to create the optimal strategy, resulting in a safe and successful surgery.

Another important point to consider preoperatively is the possibility of obstruction of the tracheal bronchus by the tracheal cuff during anesthesia [14]. In most patients with a tracheal bronchus, a left-sided DLT can successfully achieve one-lung ventilation without obstructing the tracheal bronchus because most tracheal bronchi originate from within 2 cm above the carina [2]. In the rare cases in which a tracheal bronchus branches from more than 2 cm above the carina [15], a bronchus blocker may be used instead of a left-sided DLT so that the tracheal cuff does not block the tracheal bronchus. A retrospective cohort study of 77 patients with a tracheal bronchus showed that seven (9.1%) patients had a tracheal bronchus branching from more than 2 cm above the carina with a normal diameter of the distal trachea [2]. Thus, it is important to measure the distance between the carina and the origin of the tracheal bronchus to ensure that the appropriate anesthetic techniques are used [16]. In the present case, the tracheal bronchus originated less than 2 cm above the carina; therefore, a left-sided DLT achieved successful one-lung ventilation, contributing to successful VATS segmentectomy.

## 4 Conclusion

To perform successful thoracic surgery in a patient with a tracheal bronchus, the surgeon must be aware of any accompanying anatomical anomalies, especially regarding the pulmonary venous return. Moreover, to attain optimal anesthesia, it is important to identify the origin of the tracheal bronchus.

The following is the supplementary data related to this article.

## Consent

Written informed consent was obtained from the patient for publication of this case report and accompanying images. A copy of the written consent is available for review by the Editor-in-Chief of this journal on request.

## Provenance and peer review

Not commissioned, externally peer-reviewed.

## Ethical approval

Not required.

## Funding

None.

## Guarantor

Takaki Akamine

## Research registration number

Not applicable.

## CRediT authorship contribution statement

TA wrote the manuscript. TA, TK, YS and HY participated in the surgery. TK and NM assisted in the preparation of this report. All authors read and approved the final manuscript.

## Declaration of competing interest

The authors have no conflicts of interest to declare.
